# Dynamic expression of lamin B1 during adult neurogenesis in the vertebrate brain

**DOI:** 10.1002/dvdy.70023

**Published:** 2025-04-11

**Authors:** Diana Zhilina, Lizbeth A. Bolaños Castro, Juan Sebastian Eguiguren, Sara Zocher, Anne Karasinsky, Dimitri Widmer, Alexandre Espinós, Victor Borrell, Michael Brand, Kyoko Miura, Oliver Zierau, Maximina H. Yun, Tomohisa Toda

**Affiliations:** ^1^ Nuclear Architecture in Neural Plasticity and Aging Laboratory German Center for Neurodegenerative Diseases (DZNE) Dresden Germany; ^2^ Technische Universität Dresden CRTD/Center for Regenerative Therapies Dresden Dresden Germany; ^3^ Zoo Dresden GmbH Dresden Germany; ^4^ Instituto de Neurociencias, CSIC‐UMH Sant Joan d'Alacant Spain; ^5^ Department of Aging and Longevity Research Faculty of Life Sciences, Kumamoto University Kumamoto Japan; ^6^ Environmental Monitoring & Endocrinology, Faculty of Biology Technische Universität Dresden Dresden Germany; ^7^ Physics of Life Excellence Cluster Dresden Dresden Germany; ^8^ Laboratory of Neural Epigenomics, Institute for Medical Physics and Microtissue‐Engineering, Faculty of Medicine Friedrich‐Alexander‐Universität Erlangen‐Nürnberg Erlangen Germany; ^9^ Max‐Planck‐Zentrum für Physik und Medizin Erlangen Germany

**Keywords:** adult neurogenesis, lamin B1, nuclear lamina

## Abstract

**Background:**

In mammals, specific brain regions such as the dentate gyrus (DG) of the hippocampus and the subventricular zone (SVZ) of the lateral ventricles harbor adult neural stem/progenitor cells (ANSPCs) that give rise to new neurons and contribute to structural and functional brain plasticity. In contrast, other vertebrates such as salamanders and zebrafish exhibit a widely distributed neurogenic niches throughout the brain, suggesting a greater neurogenic capacity in adulthood. However, the mechanisms underlying this divergence in neurogenic potential among vertebrates remain elusive. To address this, we examined the expression dynamics of a critical epigenetic regulator for the long‐term maintenance of murine ANSPCs, lamin B1, during adult neurogenesis across the vertebrate spectrum.

**Results:**

Lamin B1 expression patterns during adult neurogenesis are conserved among mammals including mouse, naked mole‐rat, and ferret. However, these patterns differ between mammals and anamniotes. In mammals, neural stem cells and neuroblasts exhibited higher lamin B1 levels, and differentiated neurons possessed lower lamin B1 levels. On the other hand, anamniotes showed the opposite patterns of lamin B1 expression, with higher levels in neurons compared to stem cells.

**Conclusions:**

Our study shows that the lamin B1 expression pattern during adult neurogenesis differs between species, and that changes in lamin B1 protein sequence may contribute to the differences in lamin B1 expression patterns. This study highlights potential differences in cell‐autonomous epigenetic regulation in the maintenance of ANSPC pools in the adult brain among species.

## Key Findings

- Lamin B1 expression patterns during adult neurogenesis are conserved among mammals including mouse, naked mole‐rat, and ferret. However, these patterns differ between mammals and anamniotes. This study highlights potential differences in cell‐autonomous epigenetic regulation in the maintenance of ANSPC pools in the adult brain among species.

## 1 INTRODUCTION

Adult neurogenesis is the process of formation and integration of new neurons into existing neural circuits that occurs under both normal physiological and pathological conditions.^1^, ^2^ This phenomenon is evolutionarily conserved in the forebrain across diverse vertebrates including mammals, birds, reptiles, amphibians, and fish.^3^, ^4^ During adult neurogenesis, adult neural stem/progenitor cells (ANSPCs) give rise to neurons and glial cells that play a critical role in maintaining brain function or contribute to the continuous growth of neural tissues throughout the lifespan. ANSPCs exhibit several unique cellular and molecular properties compared to neurogenesis during development. These include an extended cell cycle length, the ability to enter a quiescent state, restricted multipotency, and distinct transcriptomic programs that govern these cellular processes.^5^ In addition, comparative studies among vertebrates revealed significant differences in the rate, anatomical extent, and reparative capacity of adult neurogenesis from non‐mammalian vertebrates to mammals.^3^, ^6^, ^7^, ^8^, ^9^

In the case of mammals, under physiological conditions, adult neurogenesis is mainly restricted to two brain regions: the subventricular zone (SVZ) of the lateral ventricles (LV), from which newly generated neurons migrate to the olfactory bulb (OB),^10^ and the dentate gyrus (DG) of the hippocampus, where newborn neurons are generated locally within the subgranular zone (SGZ) of DG.^11^ Accumulating evidence suggests that adult neurogenesis contributes to structural and functional plasticity in the OB and DG, and the reduction of adult neurogenesis by aging or neuropathology affects cognition and mood regulation.^2^ In particular, the capability of adult neurogenesis decreases during aging, presumably due to both intrinsic changes of ANSPCs and extrinsic changes within the neurogenic niche.^12^, ^13^, ^14^, ^15^, ^16^, ^17^, ^18^ Furthermore, enhancing adult neurogenesis can ameliorate age‐related cognitive decline, at least in part.^19^, ^20^, ^21^ Therefore, it has been of great interest to understand the mechanisms underlying the long‐term maintenance of ANSPCs and adult neurogenesis in the mammalian brain.

In contrast to mammals, anamniotes such as salamanders and fish exhibit special progenitor cells with distinct neurogenic capability and localization in the adult brain. These radial glia‐like cells (RGLs) span the entire width of the adult brain,^6^, ^22^, ^23^ and spinal cord.^24^, ^25^ RGLs express astrocyte markers such as glial fibrillary acidic protein (GFAP), glutamine synthase, and aquaporin 4, making them similar to radial glia, the progenitor cells in the developing mammalian central nervous system (CNS).^26^, ^27^, ^28^ RGLs also exhibit distinct ependymal features; for instance, in the zebrafish spinal cord, they contribute to the ependyma.^29^ These RGLs remain active in multiple brain zones throughout the life of the organism and continually contribute to growth.^6^ Thus, anamniotes maintain the higher neurogenic capability in most adult brain regions. Therefore, the comparative study of adult neurogenesis in different vertebrates will not only deepen our understanding of conserved mechanisms in adult neurogenesis, but may also provide a clue to elucidate the mechanisms underlying the different capacity of adult neurogenesis among vertebrates.

One of the emerging mechanisms in the regulation of adult neurogenesis is epigenetic regulation. To maintain the neurogenic capability of ANSCs in the long term, genetic programs for the maintenance of ANSCs must be tightly regulated. In this regard, recent studies in rodent models have shown that nuclear structural proteins such as nuclear lamins and nuclear pore complex proteins (nucleoporins) play a critical role in maintaining epigenetic programs in ANSCs.^30^, ^31^, ^32^ Nuclear lamins are type V intermediate filament proteins located beneath the nuclear lamina, which is a dense filamentous or meshwork structure underlying the inner nuclear membrane.^33^, ^34^ Most invertebrates have one lamin gene, while vertebrates have four (*lmna, lmnb1, lmnb2* and *LIII*). Note that the *LIII* gene is lost in placental mammals and marsupials, but remainings are conserved in other vertebrates.^35^, ^36^, ^37^ The nuclear lamina is crucial for various functions, including heterochromatin organization, transcriptional regulation, maintenance of nuclear structure and shape, nuclear assembly and disassembly, and other nuclear processes.^33^, ^34^ Mutations in lamin genes are associated with several human diseases, known as “laminopathies” or “nuclear envelopathies,” including cardiac and muscular dystrophies, lipodystrophy, and premature aging syndromes.^38^ In the central nervous system, lamin Bs are dominant since the expression of lamin A is repressed by miR‐9.^39^ These nuclear structural proteins can directly or indirectly interact with chromatin, and also recruit other transcriptional and epigenetic factors to tightly regulate lineage‐specific genetic programs.^32^, ^40^ Importantly, some of these proteins are extremely stable and do not turn over frequently.^41^, ^42^ Therefore, the long‐lived nuclear structural protein‐mediated epigenetic regulation could be a robust mechanism to maintain cell type‐specific epigenetic programs in long‐lived cells such as neurons or ANSPCs/RGLs. Indeed, the expression levels of lamin B1 dynamically change during adult neurogenesis in the mouse DG, and lamin B1 plays a crucial role in preventing premature differentiation of ANSPCs and regulating their maintenance.^30^, ^31^ Lamin B1 levels decrease with age, and loss of lamin B1 leads to the depletion of ANSCs and adult neurogenesis.^30^, ^31^, ^43^ Thus, lamin B1 contributes to maintaining neurogenic capacity in the adult rodent hippocampus, and the dynamic regulation of lamin B1 expression likely contributes to proper adult neurogenesis. These observations prompted us to compare the expression patterns of lamin B1 in different species during the process of adult neurogenesis.

To infer the role of lamin B1 in adult neurogenesis among different species, we compared lamin B1 expression patterns in other species including three different mammals: mouse (*Mus musculus*), naked mole‐rat (*Heterocephalus glaber*), and ferret (*Mustela putorius furo*) and three different anamniotes: axolotl (*Ambystoma mexicanum*), the Iberian ribbed newt (*Pleurodeles waltl*), and zebrafish (*Danio rerio*). We hypothesize that the dynamics of lamin B1 expression in neural lineage cells associate with adult neurogenesis potential. By comparing lamin B1 expression patterns during adult neurogenesis among different vertebrate models, we aimed to uncover conserved patterns or species‐specific adaptations of lamin B1 expression that may underlie species‐specific adult neurogenesis features. Through our analyses, we found that the patterns of lamin B1 expression during adult neurogenesis differ between mammals and anamniotes, and the patterns of lamin B1 correlate with the overall similarity of lamin B1 protein sequences. Our observation suggests that the role of lamin B1 in adult neurogenesis may differ between species, and the distinct expression patterns of epigenetic machinery may contribute to the distinct neurogenic capacity.

## 2 RESULTS

### 2.1 Expression pattern of lamin B1 during adult neurogenesis in mice

**FIGURE 1:**
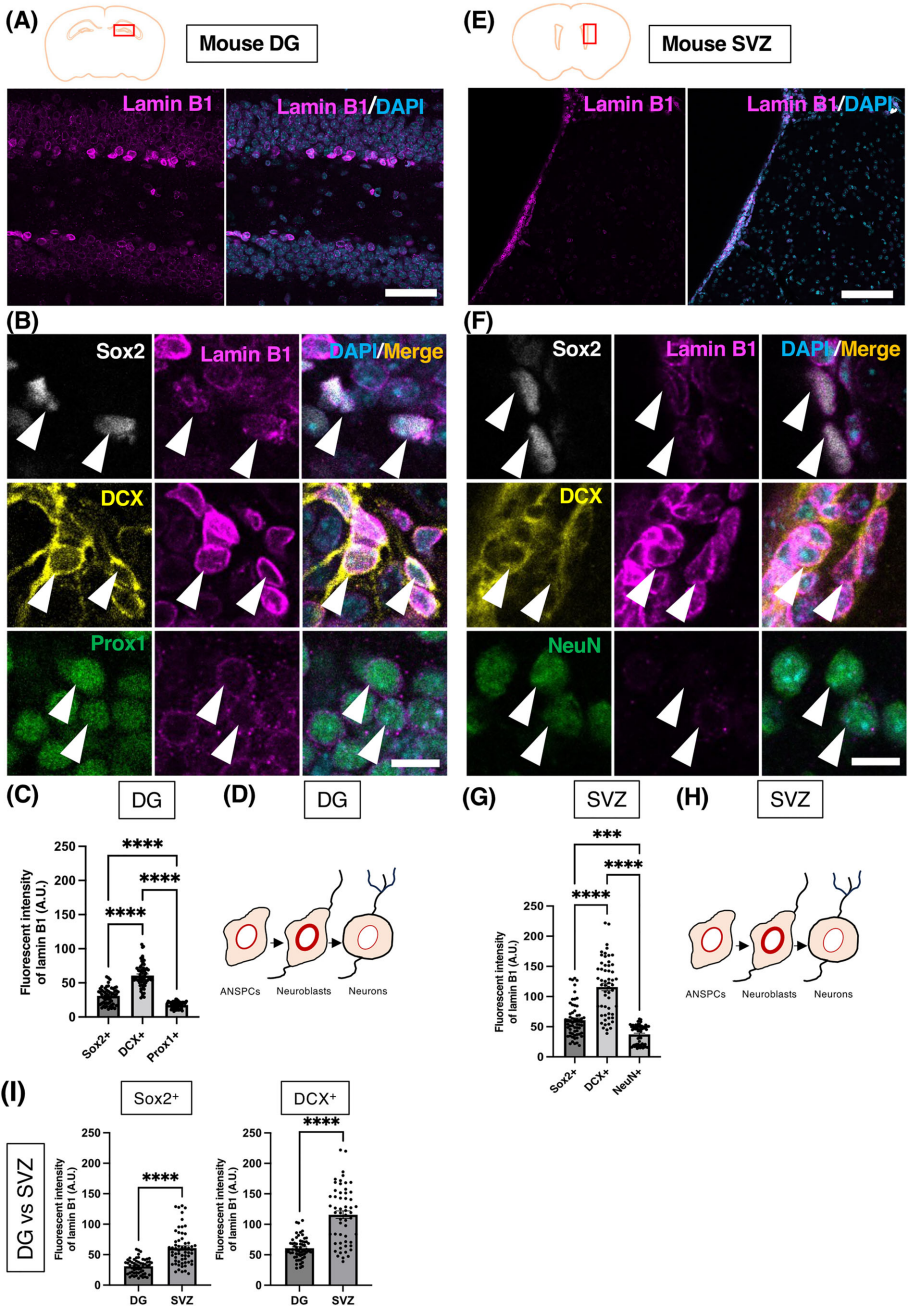
Lamin B1 expression patterns in the mouse DG and SVZ. (A, E) Confocal images of the adult mouse DG (A) and the SVZ (E) stained with lamin B1 (magenta) and DAPI (blue). (B, F) IHC staining for the mouse DG (B) and SVZ (F) depicting lamin B1 expression in Sox2^+^ ANSPCs (white), DCX^+^ neuroblasts (yellow), and Prox1^+^ or NeuN^+^ neurons (green). Arrowheads point at respective cell types. (C, G) Quantification of lamin B1 protein levels in the DG (C), and the SVZ (G) using immunofluorescent signals. (D, H) A schema for the changes in lamin B1 levels during adult neurogenesis in DG (D) and SVZ (H) of mouse. (I) Relative comparison of lamin B1 expression in DG and SVZ of mouse. A.U., arbitrary units. Bar graphs show mean ± SEM. Scale bars, 100 μm in (A, E), 10 μm in (B, F), *n* = 3 animals.

**FIGURE 2:**
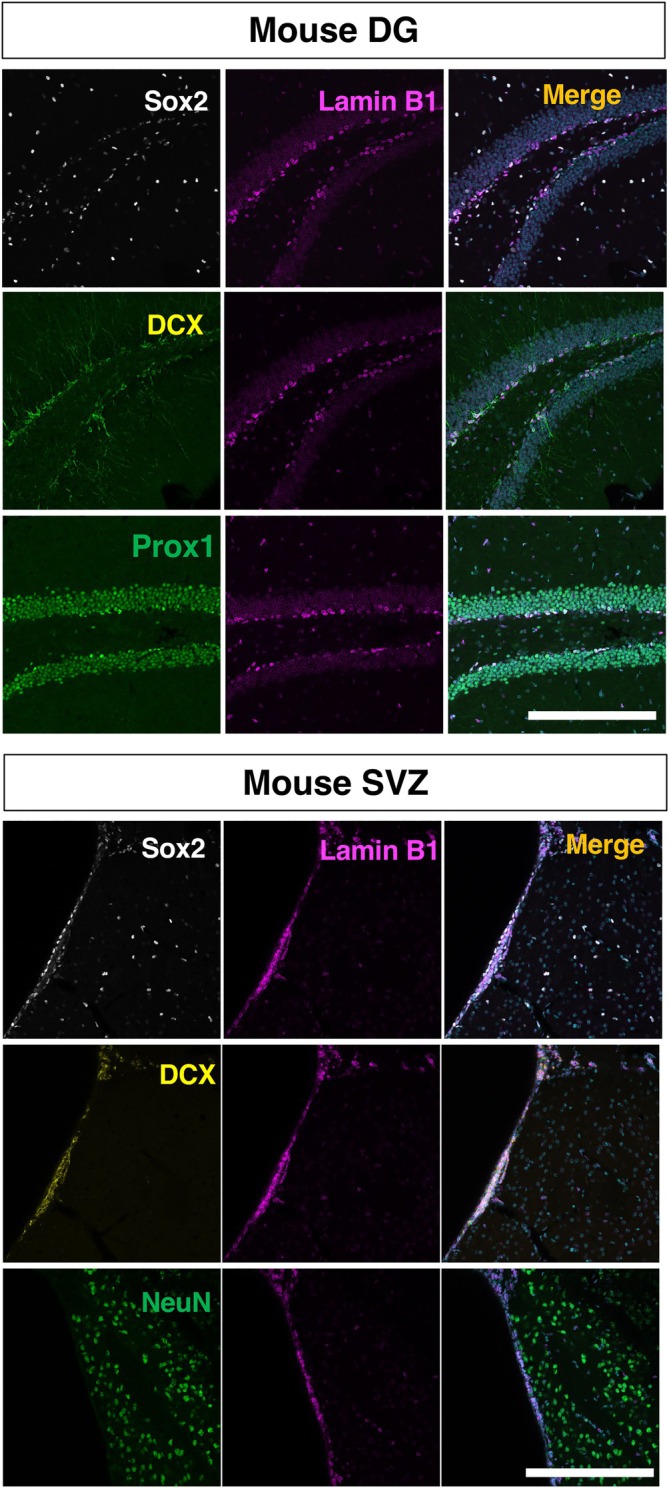
The overview of lamin B1 expression in the mouse DG and SVZ counterstained with the neural stem cell marker Sox2, the neuroblast marker DCX, or the neuronal markers Prox1 or NeuN. Scale bar = 200 μm.

In the previous studies, dynamic changes in lamin B1 expression were observed during the process of adult neurogenesis in the mouse DG.^30^, ^31^ First, we investigated whether this dynamic pattern of lamin B1 expression is consistent between two different neurogenic regions in the mouse brain, the SGZ of the DG and the SVZ of the LV. To investigate the dynamics of lamin B1 during adult neurogenesis in the mouse DG, the expression levels of lamin B1 were examined in adult neural stem/progenitor cells (ANSPCs, Type1/2a, Sox2^+^) and neuroblasts (Type 3, Doublecortin [DCX]^+^) in the SGZ, as well as in Prox1^+^ dentate granule neurons in the outer granule zone (OGZ) (Figures 1 and 2). The same imaging conditions were used within the same species. Consistent with the previous observations,^30^, ^31^ the strongest signal of lamin B1 was observed in neuroblasts (60.82 ± 2.29 A.U.), followed by ANSPCs (30.85 ± 1.60 A.U.) (Figure 1A–D). In contrast, Prox1^+^ dentate granule neurons in the OGZ expressed the lowest level of lamin B1 (17.64 ± 0.64 A.U.) (Figure 1C) (****p < .0001; one‐way ANOVA followed by Tukey's multiple comparison test). Next, we examined the expression levels of lamin B1 in ANSPCs (type B1/C, Sox2^+^), neuroblasts (type A, DCX^+^) in the SVZ, and neurons in the striatum derived from the lateral ganglionic eminence (LGE) along the LV. Similar to the DG, lamin B1 in the SVZ was found to be highly expressed in neuroblasts (115.8 ± 6.20 A.U.), followed by ANSPCs (60.83 ± 3.69 A.U.) compared to NeuN^+^ neurons (37.17 ± 2.03 A.U.) (****p < 0.0001; ***p = 0.0003, one‐way ANOVA test followed by Tukey's multiple comparison test) (Figure 1E–H). These data indicate that the pattern of lamin B1 expression is consistent between two different neurogenic regions, supporting the idea that lamin B1 expression patterns might play a role in the regulation of adult neurogenesis in the mouse brain, as previously demonstrated in the DG. We also assessed whether there is a difference between the lamin B1 levels in the corresponding cell types between the DG and SVZ. Interestingly, we found overall higher levels of lamin B1 expression in ASNPCs/neuroblasts cells in the SVZ compared to those in the DG (Figure 1I) (ANSPCs, ****p < .0001 Mann–Whitney test; neuroblasts, ****p < .0001 unpaired *t*‐test). Since neurogenic capacity is higher in the SVZ,^44^ and higher levels of lamin B1 are essential to maintain ANSPCs,^30^, ^31^ higher lamin B1 in the SVZ may contribute to these properties. Furthermore, since nuclear integrity is crucial during neural migration,^45^ higher lamin B1 in type A neuroblasts may protect their nuclei during their longer migration distance from the LV to OB compared to neuroblasts in the DG.

### 2.2 Expression pattern of lamin B1 during adult neurogenesis in naked mole‐rats

**FIGURE 3:**
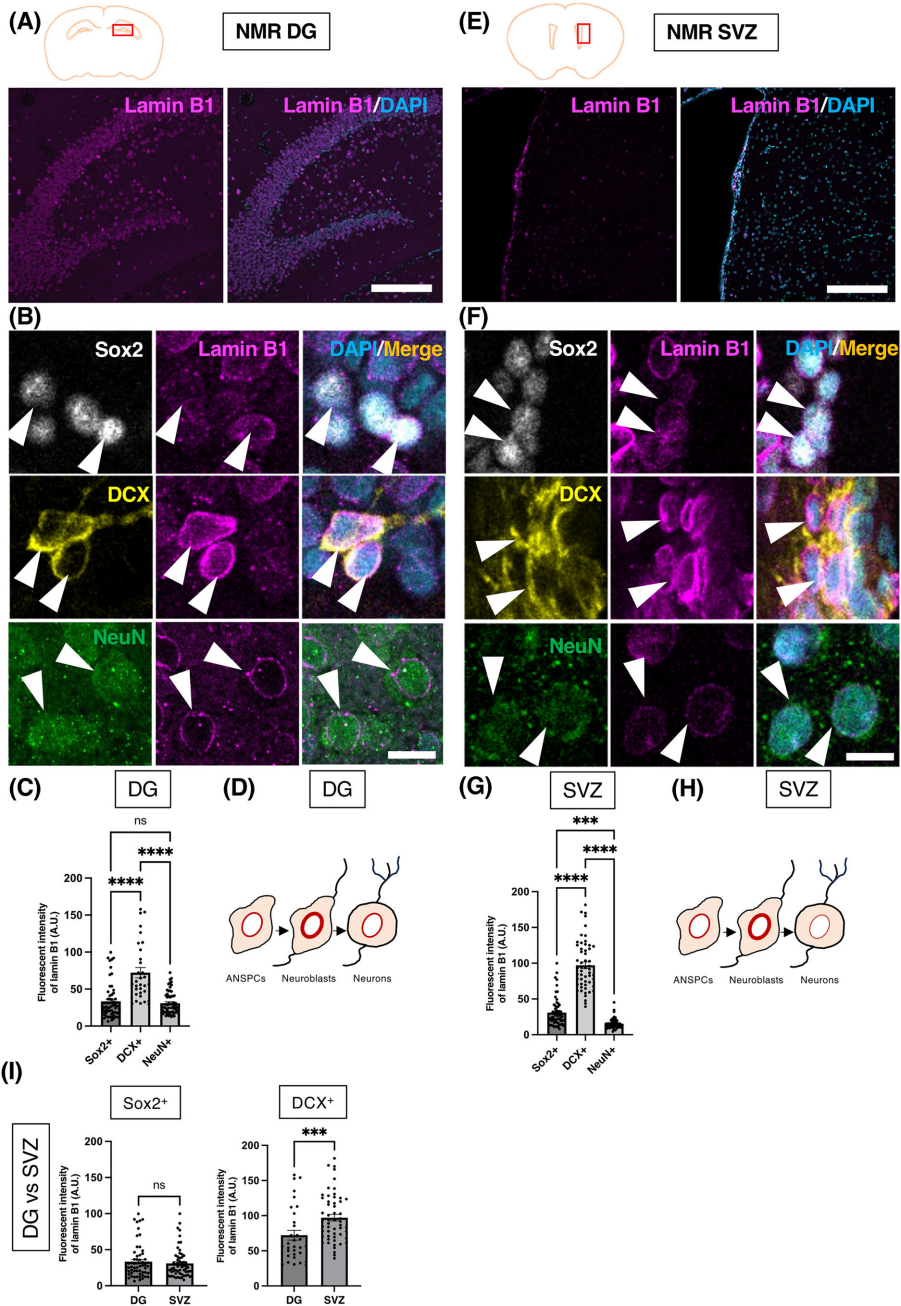
Lamin B1 expression patterns in the NMR DG and SVZ. (A, E) Confocal images of the adult NMR DG (A) and SVZ (E) stained with lamin B1 (magenta) and DAPI (blue) (B, F) IHC staining for the DG (B) and the SVZ (F) depicting lamin B1 expression in Sox2^+^ ANSPCs (white), DCX^+^ neuroblasts (yellow), and NeuN^+^ neurons (green). Arrowheads point at respective cell types (C, G) Quantification of lamin B1 protein levels in the NMR DG (C) and SVZ (G) using immunofluorescent signals (D, H). A schema for the changes in lamin B1 levels during adult neurogenesis in the DG (D) and the SVZ (H) of NMR. (I) Relative comparison of lamin B1 expression in the DG and SVZ of NMR. A.U., arbitrary units. Bar graphs show mean ± SEM. Scale bars, 200 μm in (A, E), 10 μm in (B, F), *n* = 3 animals.

**FIGURE 4:**
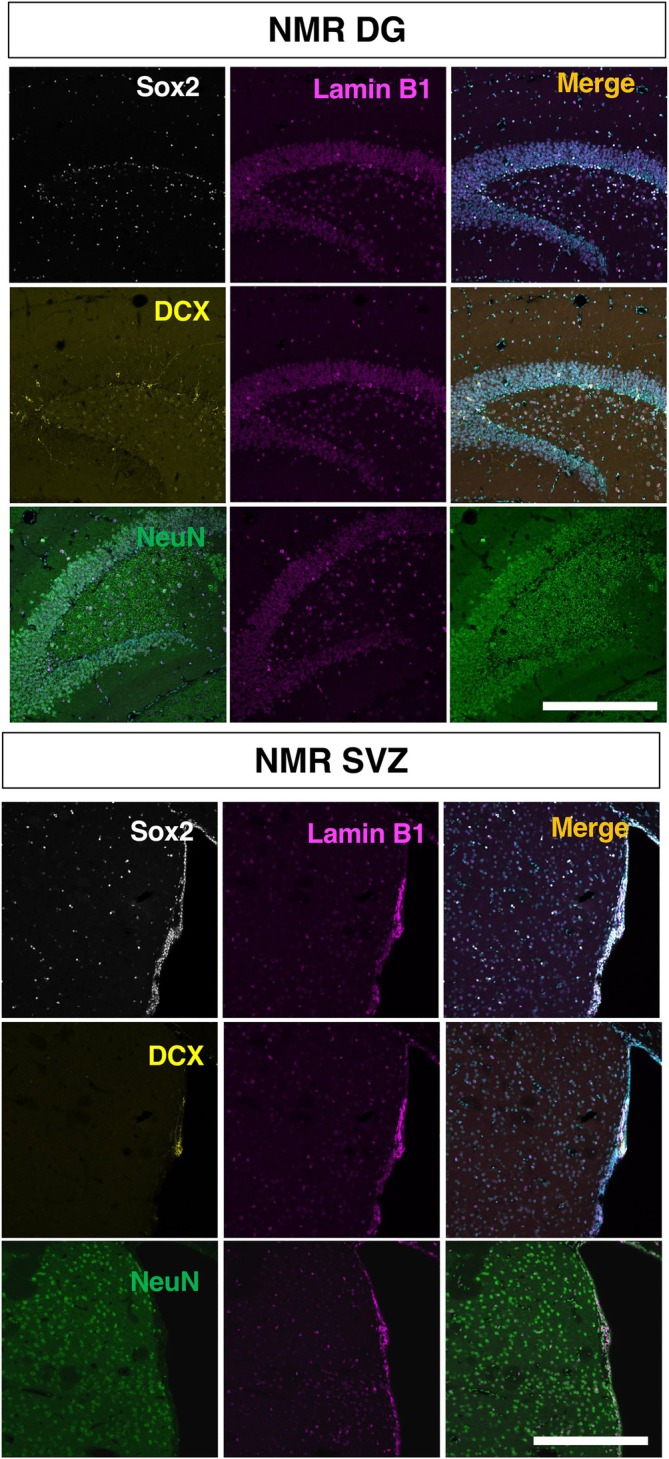
The overview of lamin B1 expression in the NMR DG and SVZ counterstained with neural stem cell marker Sox2, neuroblast marker DCX, or neuronal marker NeuN. Scale bar = 300 μm.

Having observed the consistent expression patterns of lamin B1 in the two neurogenic regions of the mouse, we then investigated whether other mammals with longer lifespans show similar patterns. The naked mole‐rat (NMR) is a remarkably long‐lived rodent species with a recorded lifespan in captivity of over 37 years,^46^ offering unique insights into neurogenesis due to its extended lifespan and slower maturation.^47^, ^48^ NMRs possess significantly reduced rates of hippocampal adult neurogenesis in comparison to mice,^49^, ^50^ and the SVZ is the major source of adult cell proliferation in NMRs.^48^ Interestingly, NMRs show an extremely protracted period of brain maturation, which may allow brain plasticity and resistance to neurodegeneration over decades of life.^50^ Similar to mice, immunohistochemistry (IHC) for lamin B1 was performed on coronal sections of adult NMR brains, and the levels of lamin B1 expression were quantified in the DG and SVZ (Figures 3 and 4).

Interestingly, despite the different rate of neurogenesis and developmental speed, the pattern of lamin B1 expression in the DG and SVZ of NMR was found to be similar to that in the mouse (Figure 3). Lamin B1 levels in the DG were found highest in DCX^+^ neuroblasts (72.21 ± 6.94 A.U.), whereas Sox2^+^ ANSPCs (33.43 ± 3.03 A.U.) and NeuN^+^ neurons (30.99 ± 1.97 A.U.) exhibited lower levels of lamin B1 (Figure 3A–D) (****p < .0001, ns not significant, one‐way ANOVA test followed by Tukey's multiple comparison test). Similarly, DCX^+^ cells showed the highest level of lamin B1 in the SVZ (97.14 ± 4.62 A.U.), followed by Sox2^+^ ANSPCs (30.86 ± 2.59 A.U.) and NeuN^+^ neurons (15.32 ± 0.88 A.U.) (Figure 3E–H) (****p < .0001, ***p = .0008; one‐way ANOVA test followed by Tukey's multiple comparison test). Furthermore, the levels of lamin B1 in neuroblasts were higher in the SVZ than in the DG, consistent with mice (Figure 3I), indicating that higher levels of lamin B1 in neuroblasts of the SVZ could be critical for neurogenesis. Since the pattern of lamin B1 expression in adult neurogenesis is similar between NMRs and mice, this observation supports the idea that lamin B1 contributes to adult neurogenesis both in NMRs and mice.

### 2.3 Expression pattern of lamin B1 during adult neurogenesis in ferret

**FIGURE 5:**
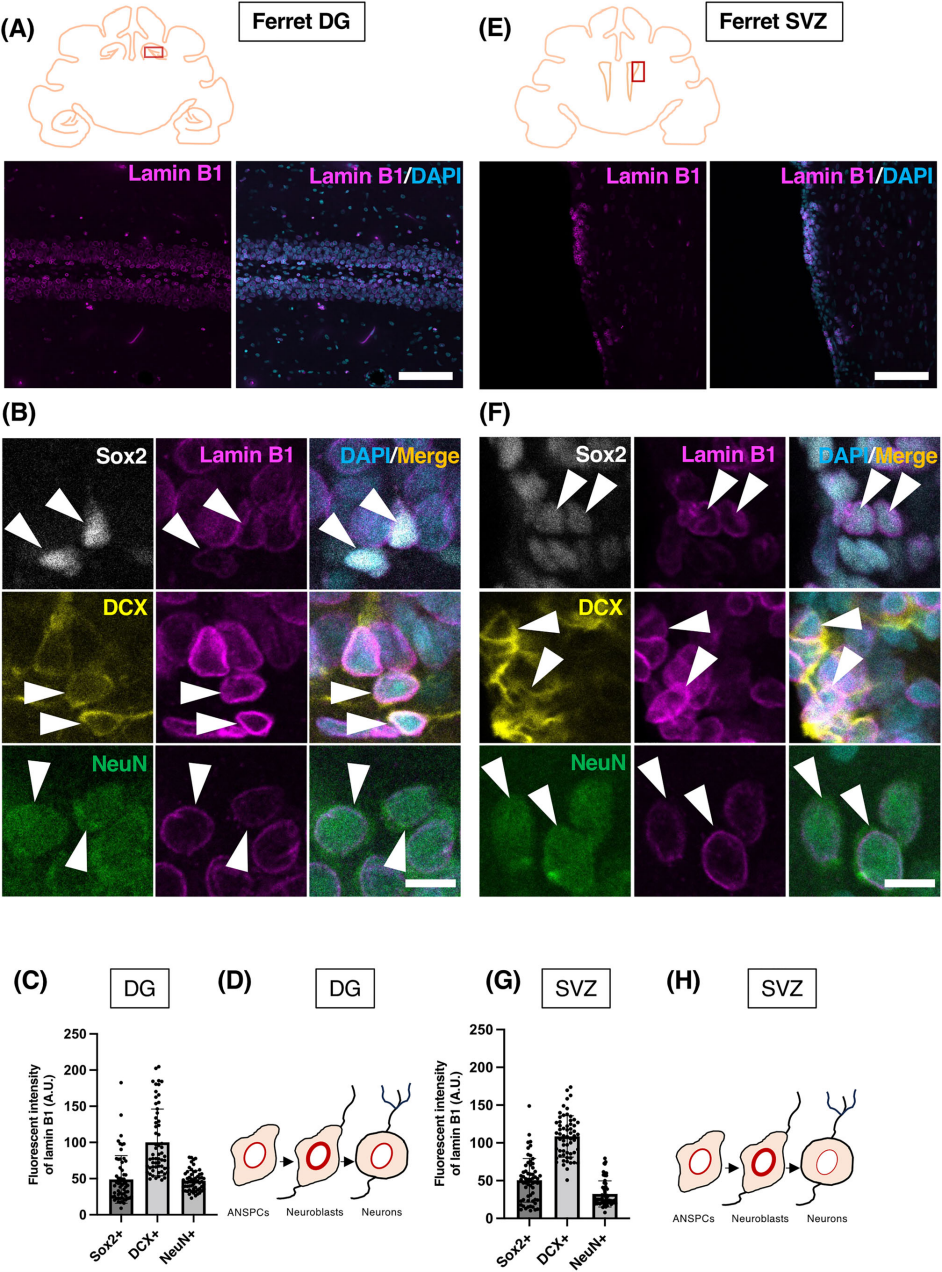
Lamin B1 expression patterns in the ferret DG and SVZ. (A, E) Confocal images of the adult ferret DG (A) and SVZ (E) stained with lamin B1 (magenta) and DAPI (blue). (B, F) IHC staining for DG (B) and SVZ (F) depicting lamin B1 expression in Sox2^+^ (white) ANSPCs, DCX^+^ (yellow) neuroblasts and NeuN^+^ (green) neurons. Arrowheads point at respective cell types. (C, G) Quantification of lamin B1 protein levels in ferret DG (C) and SVZ (G) using immunofluorescent signals. (D, H) A schema for lamin B1 dynamics during adult neurogenesis in different neural lineage cells of DG (D) and SVZ (H) of ferret. (I) Relative comparison of lamin B1 expression in DG and SVZ of ferret. A.U., arbitrary units. Bar graphs show mean ± SEM. Scale bars, 100 μm in (A, E), 10 μm in (B, F), *n* = 1 animal.

**FIGURE 6:**
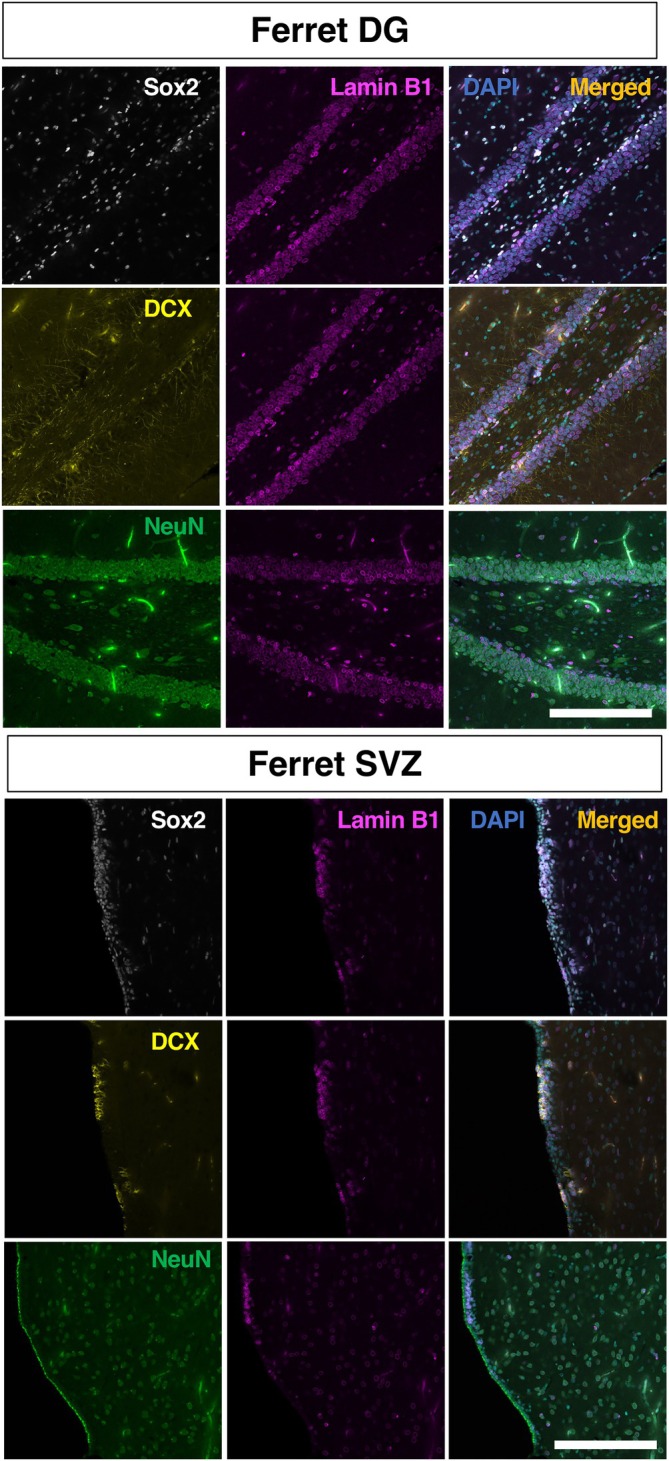
The overview of lamin B1 expression in the ferret DG and SVZ region counterstained with the neural stem cell marker Sox2, the neuroblast marker DCX, or the neuronal marker NeuN. Scale bar = 200 um.

In addition to rodents, we aimed to address a species from a different branch of the phylogenetic tree. The ferret is a mammal from the order of carnivores and has been shown to exhibit adult neurogenesis in both canonical mammalian neurogenic niches—DG and SVZ (Figures 5 and 6).^48^ Due to limited sample availability, we analyzed only one sample. Analysis of lamin B1 intensities in the DG and SVZ revealed a similar pattern to that observed in rodents. Thus, consistent with both mice and NMRs, the highest lamin B1 expression was found in DCX^+^ neuroblasts (Figure 5C–H). Therefore, we concluded that the lamin B1 expression pattern during adult neurogenesis is generally conserved in the studied mammalian species.

### 2.4 Expression pattern of lamin B1 during adult neurogenesis in salamander

In the three mammalian species, we observed the consistent pattern of lamin B1 expression in two adult neurogenic regions. This observation prompted us to address the pattern of lamin B1 expression in salamanders, which exhibit widespread proliferation throughout the adult brain.^22^, ^51^, ^52^, ^53^ We examined two salamander species known for post‐embryonic neurogenesis in multiple brain regions: the axolotl (*Ambystoma mexicanum*) and the Iberian Ribbed Newt (*Pleurodeles waltl*), a salamander species with a true adult (post‐metamorphic) stage.

**FIGURE 7:**
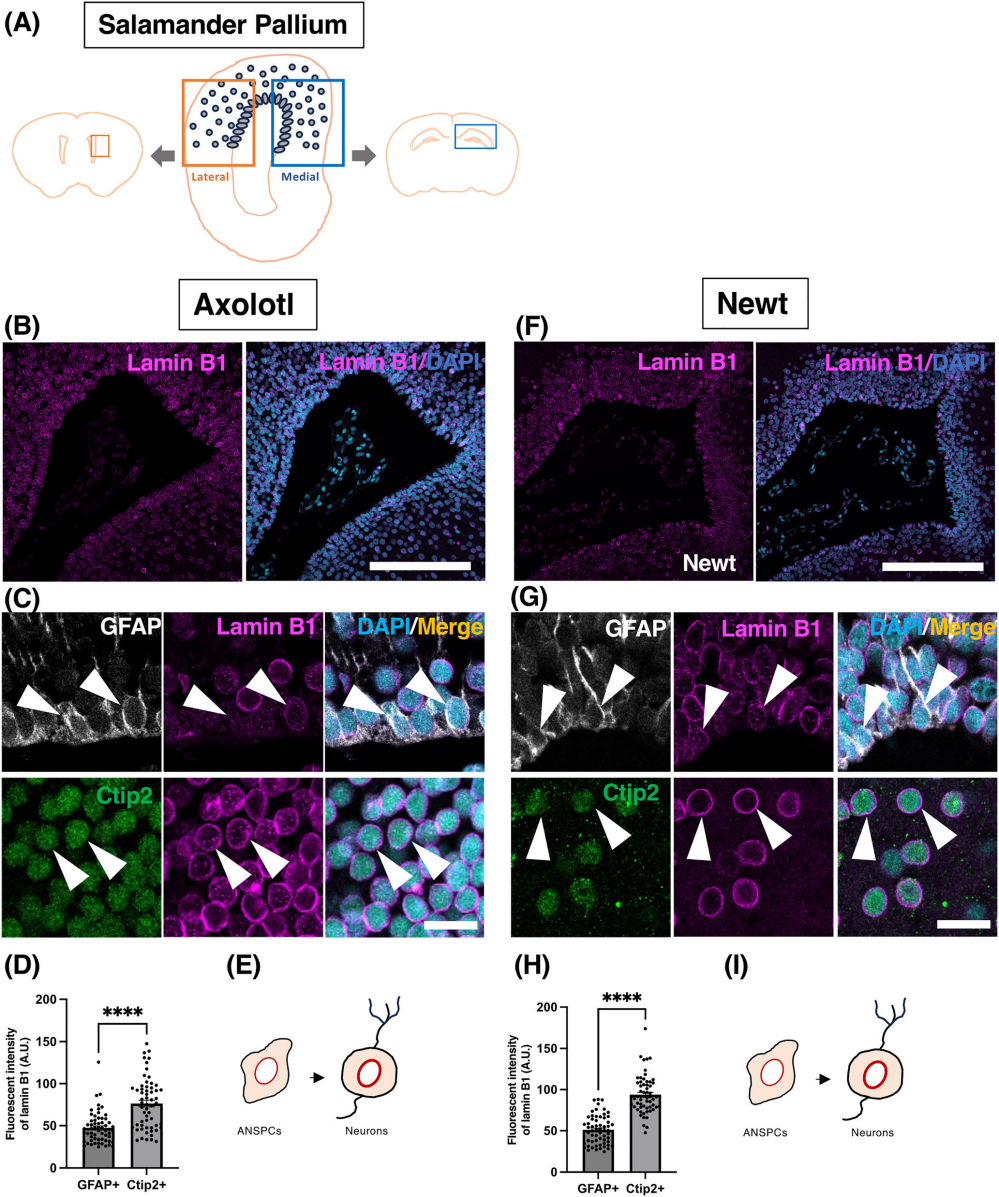
Lamin B1 expression patterns in the salamander pallium. (A) A schema for regions of interest representing respective regions for analyses in the mammalian brain. (B, F) Confocal images of the mature axolotl pallium(B) and newt pallium (F) stained with lamin B1 (magenta) and DAPI (blue). (C, G) IHC staining for axolotl (C) and newt (G) showing lamin B1 expression in GFAP^+^ ANSPCs (white) and Ctip2^+^ pallium neurons (green). Arrowheads point at respective cell types. (D, H) Quantification of lamin B1 protein levels in the axolotl pallium (D) and the newt pallium (H) using immunofluorescent signals (E, I). A schema depicting the lamin B1 dynamics in neural lineage cells in the axolotl pallium (E) and the newt pallium (I) A.U., arbitrary units. Bar graphs show mean ± SEM. Scale bars, 300 μm in (A, E), 25 μm in (B, F), *n* = 3 animals per species.

**FIGURE 8:**
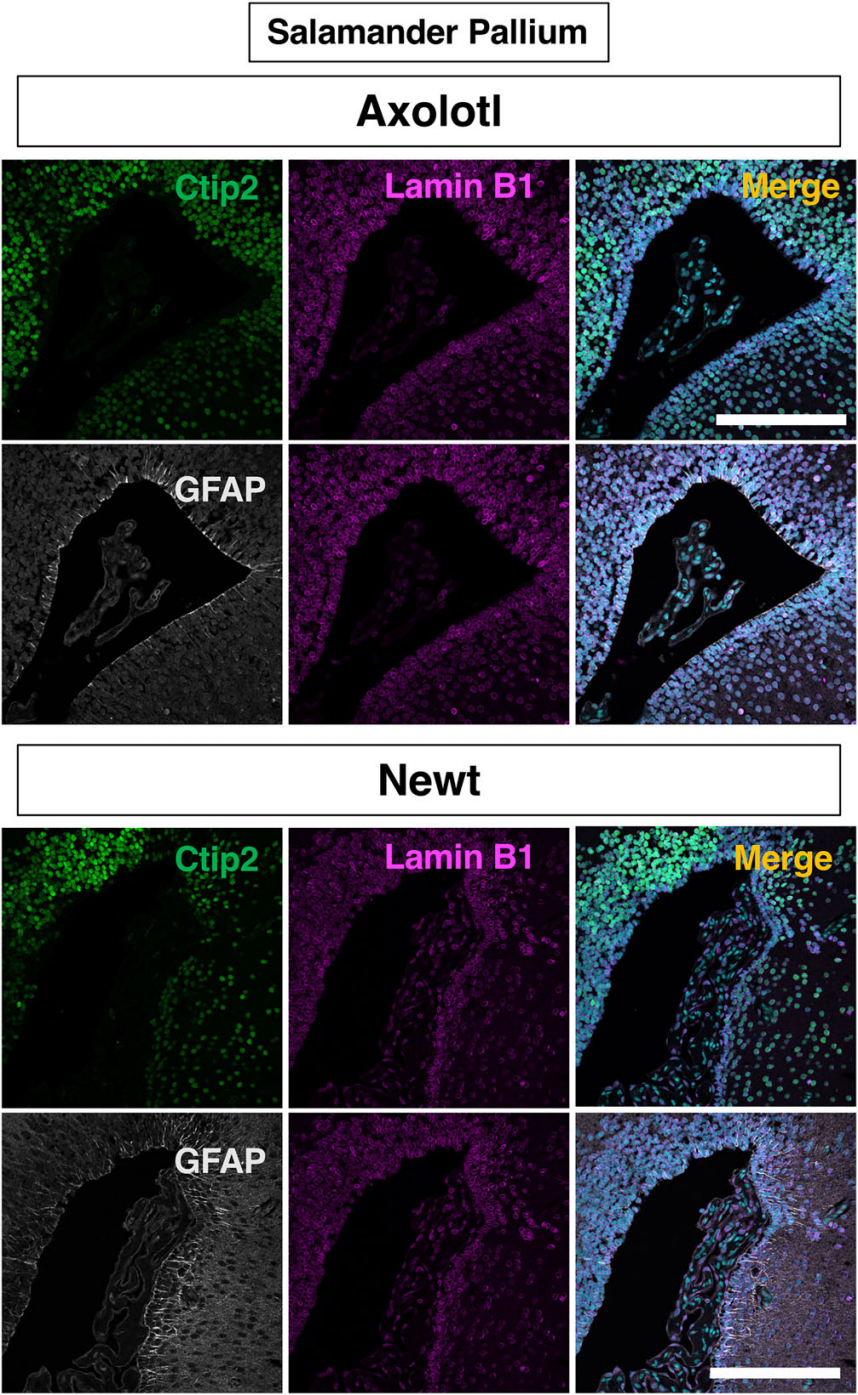
The overview of lamin B1 expression in the axolotl and newt pallium counterstained with the neural stem cell marker GFAP and the neuronal marker Ctip2. Scale bar = 300 μm.

To compare the corresponding brain regions between mammals and salamanders, we focused on the telencephalon of salamanders, as previous studies have demonstrated the molecular similarity between salamander pallium neurons and amniote cortical/hippocampal neurons.^54^, ^55^, ^56^ We examined both the medial and lateral pallium and observed a consistent pattern of lamin B1 expression (Figures 7A and 8), and therefore combined the data from the medial and lateral pallium. Due to technical reasons, we were unable to identify neuroblasts in the salamander brain, so we focused on GFAP^+^ RGLs, which we refer to as ANSPCs in analogy to mammals, and Ctip2^+^ neurons in the pallial region. IHC revealed significantly higher levels of lamin B1 in neurons in comparison to the ANSPCs both in axolotl and newt (Figure 7B–I), which is in contrast to what we found in mammals (****p < .0001; Mann–Whitney test, axolotls, 78.31 ± 4.30 A.U. in neurons vs. 47.44 ± 2.35 A.U. in ANSPCs; newts, 93.86 ± 2.96 A.U. in neurons vs. 51.08 ± 2.22 A.U. in ANSPCs; *p* < .0001). This indicates that cell type‐specific lamin B1 expression differs between mammals and salamanders and that lamin B1 may have a different function in adult neurogenesis between them. Since both salamanders exhibit higher lamin B1 levels in neurons compared to ANSPCs, lamin B1 may have more important roles in post‐mitotic neurons in salamanders, such as the maintenance of heterochromatin in their giant genomes.

### 2.5 Expression pattern of lamin B1 during adult neurogenesis in zebrafish

**FIGURE 9:**
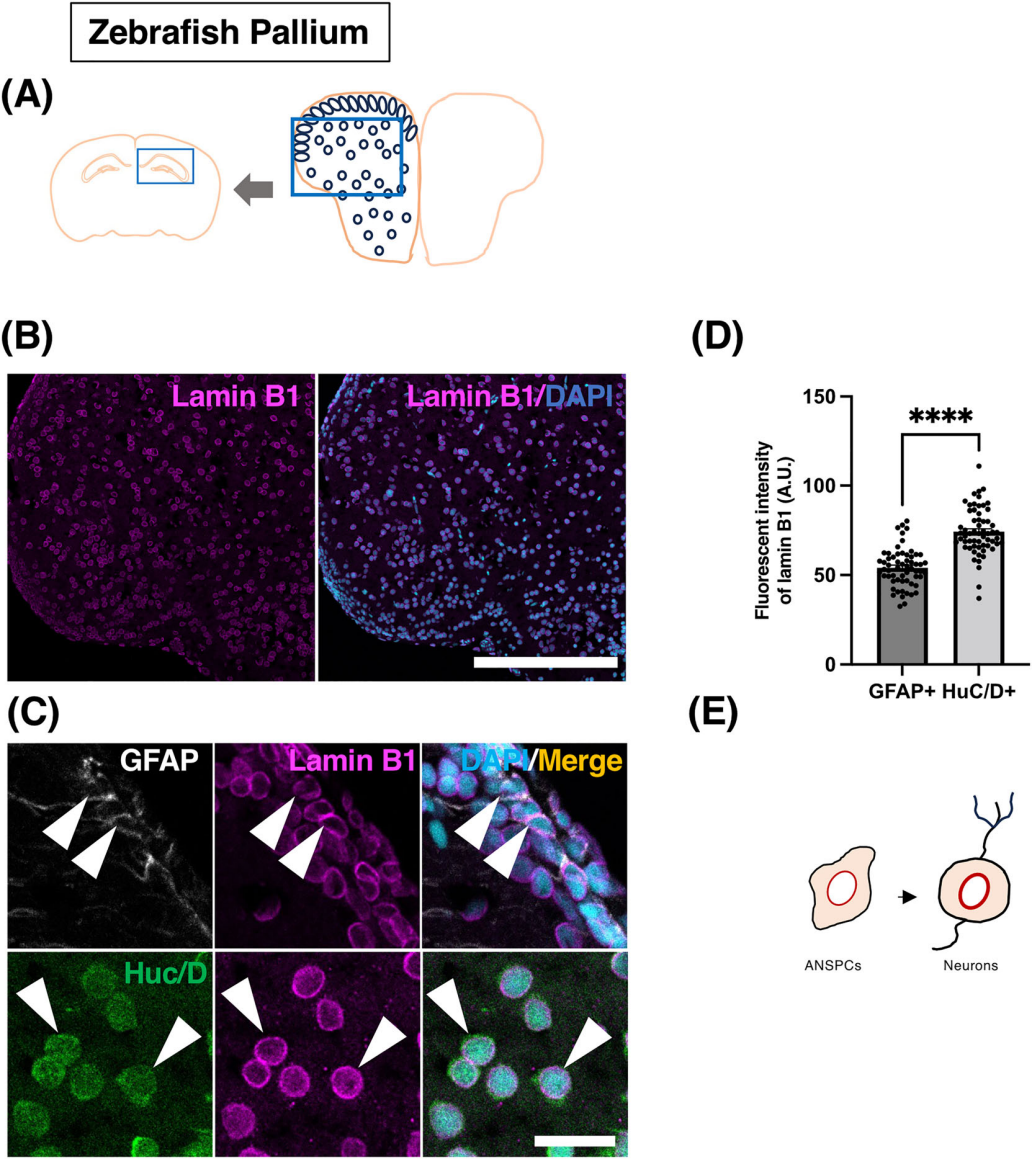
Lamin B1 expression patterns in the zebrafish pallium. (A) A schema of regions of interest for analysis representing a corresponding region for analysis in the mammalian brain. (B) Confocal images of dorso‐lateral pallium of adult zebrafish stained with lamin B1 (magenta) and DAPI (blue). (C) IHC staining for zebrafish demonstrating lamin B1 expression in GFAP^+^ ANSPCs (white), and HuC/D^+^ neurons (green). Arrowheads point at respective cell types. (D) Quantification of lamin B1 protein levels in the zebrafish dorso‐lateral pallium using immunofluorescent signals. (E) A schema depicting the lamin B1 expression pattern in neural lineage cells in the zebrafish dorso‐lateral pallium. A.U., arbitrary units. Bar graphs show mean ± SEM. Scale bars, 200 μm in (A), 20 μm in (B), *n* = 3 animals.

**FIGURE 10:**
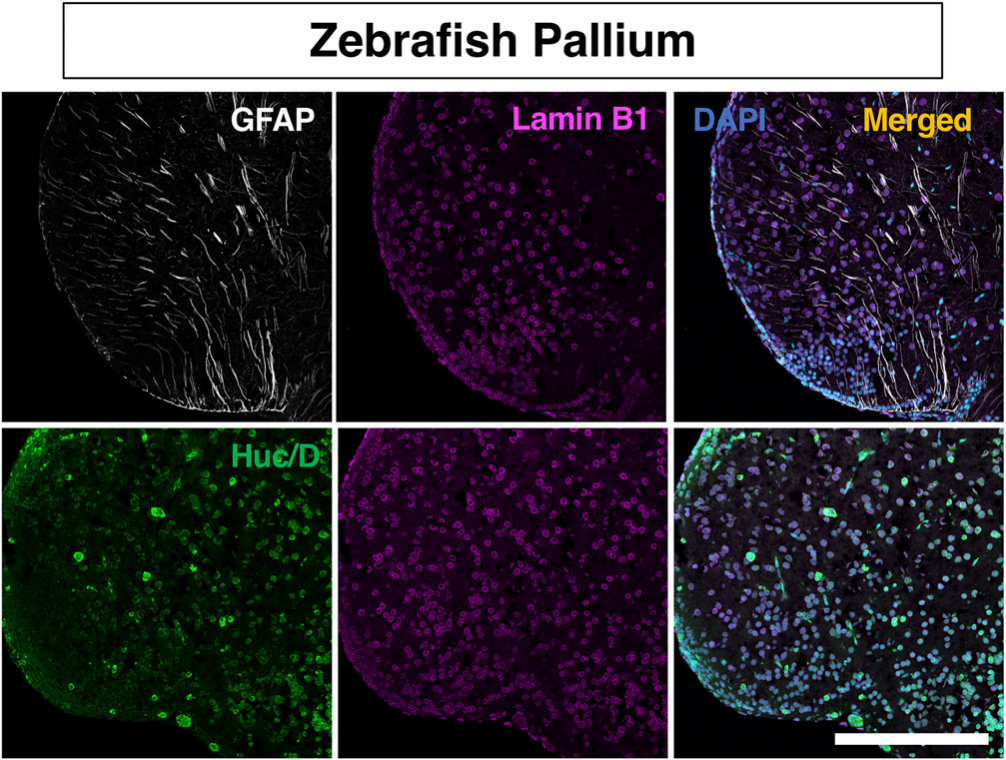
The overview of lamin B1 expression in the zebrafish pallium counterstained with neural stem cell marker GFAP and neuronal marker HuC/D. Scale bar = 200 μm.

Finally, we examined the pattern of lamin B1 expression in the teleost vertebrate, zebrafish (*Danio rerio*). Zebrafish possess a substantial ANSPC population located along the ventricular surface of the dorsal telencephalon (pallium), regions analogous to adult neurogenic niches observed in the mammalian brain.^6^, ^57^ In zebrafish, ongoing proliferation and neurogenesis persist throughout life, facilitating continuous brain growth and enabling exceptional regenerative potential.^6^, ^9^, ^58^ ANSPCs in the ventricular zone are similar to mammalian neural stem cells, exhibiting features such as the expression of GFAP, a relatively quiescent state, differentiation into amplifying progenitors, and continuous neuronal generation throughout the lifespan.^6^, ^59^ We focused our analysis on the dorso‐lateral telencephalon (pallium), likely homologous to the mammalian hippocampus (Figures 9A and 10), as previously described,^6^, ^60^ and used GFAP to identify ANSPCs of zebrafish and HuC/D as a marker of neurons.^6^ We observed that the levels of lamin B1 in neurons (74.29 ± 1.69 A.U.) were significantly higher than in ANSPCs (54.12 ± 1.38 A.U.) in the zebrafish dorso‐lateral pallium (Figure 9B–E) (****p < .0001; Mann–Whitney test). This pattern of lamin B1 expression during neurogenesis is consistent with the pattern we observed in salamanders (Figure 7). These data suggest that the patterns of lamin B1 during adult neurogenesis are conserved among anamniotes, and they are distinct from those in mammals. In the future, to fully understand the dynamics of lamin B1 expression during adult neurogenesis in anamniotes, it would be important to determine the levels of lamin B1 in neuroblasts using cell type‐specific markers.

### 2.6 Evolutional comparison of lamin B1 protein sequences

Since the expression patterns of lamin B1 during adult neurogenesis are distinct between mammals and anamniotes, we wondered if changes in lamin B1 protein structure, which impact protein function, are associated with changes in lamin B1 expression patterns. To this end, we assessed the conservation of lamin B1 protein structure by comparing amino acid sequence identity and similarity values as well as the conservation of post‐translational modifications (PTMs), which are critical modifications to modulate lamin B1 stability and function.^61^

**FIGURE 11:**
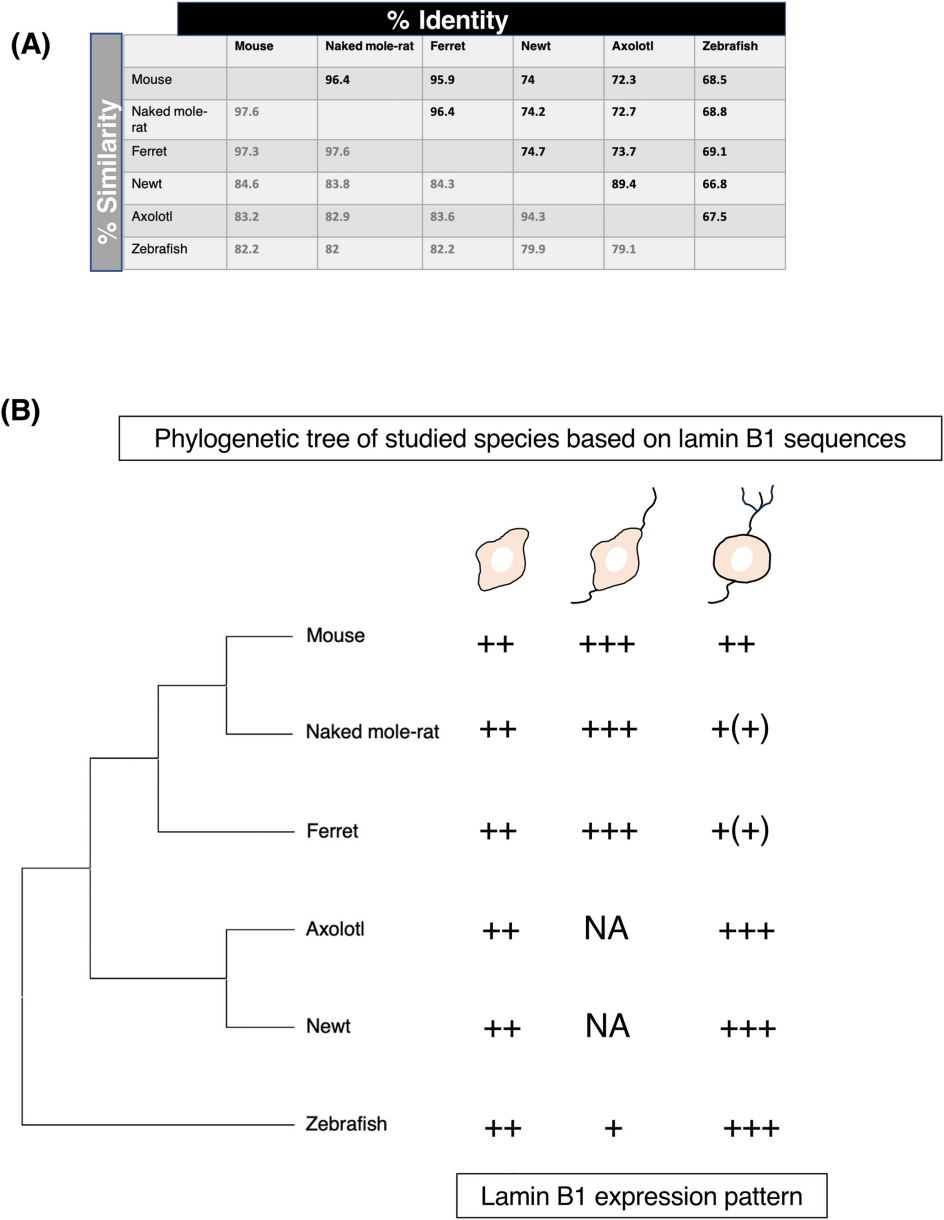
Correlation between lamin B1 protein sequence similarity and its expression patterns. (A) The identity and similarity values for lamin B1 protein sequences show higher conservation among mammals and lower conservation between mammals and anamniotes. (B) Summary of the results. Lamin B1 sequence identity and similarity correlate with the lamin B1 expression patterns among species.

**FIGURE 12:**
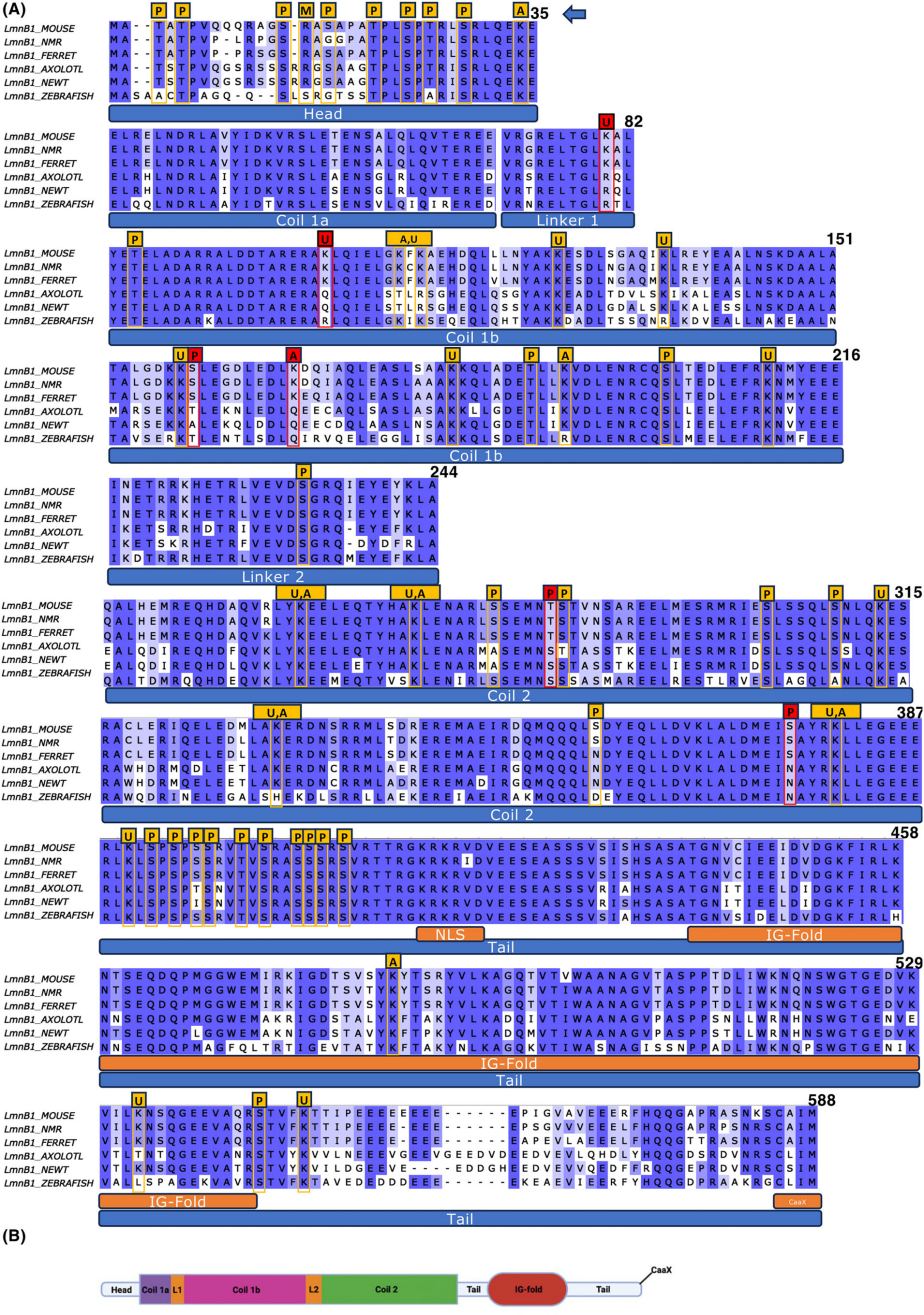
Multiple sequence alignment of lamin B1 proteins in the studied species. (A) MSA of lamin B1, depicting protein domains and motifs as well as known PTM sites (highlighted with frames), identified according to the mouse lamin B1 protein sequence (P‐phosphorylation, M‐methylation, A‐acetylation, and U‐ubiquitination). Non‐conserved potential PTM sites between mammalians and non‐mammalians are indicated in red. (B) Lamin B1 protein structure scheme, demonstrating domains, indicated in (A). The darker blue color area indicates higher similarity in lamin B1 protein sequences.

First, to make a pairwise comparisons of lamin B1 protein sequences and assess whether two sequences share a common origin, we used the Ident and Sim function of the sequence manipulation suite for pairwise comparison (http://www.bioinformatics.org/SMS/index.html), which calculates protein sequence identity and similarity. For similarity calculations, the following groups of amino acids with similar physicochemical properties or conserved functional roles were used: ILV, FWY, KRH, DE, GAS, P, C, or TNQM. These groups represent standard classifications often employed in multiple sequence alignments and are the default setting in the suite used in this analysis. Sequence identity refers to the exact match of amino acids at corresponding positions in an alignment, indicating that the same residue is present in both sequences. In contrast, sequence similarity accounts for amino acids with similar physicochemical properties, even if they are not identical. This allows for substitutions that may still maintain the structure or function of the protein, providing a broader perspective on evolutionary relationships and conservation. The analysis revealed a high conservation level among the studied mammalian species (Figure 11A), suggesting that mammalian lamin B1 protein structure is well conserved. In contrast, the identity and similarity values between mammalian and non‐mammalian species were below 75% although the general structure of the protein is conserved, with all domains and motifs present across all species (Figure 12).

Second, to find evolutionary relationships between lamin B1 sequences and lamin B1 expression patterns, multiple sequence alignment (MSA) and phylogenetic analysis were conducted (Figure 11B). The phylogenetic analysis of lamin B1 protein sequences divided the studied animal classes into two groups. One of the clusters refers to mammalian species that are more closely related to each other than to the other animal group, which consisted of anamniotes (Figure 11B). The phylogenetic analysis is correlated with the pattern of lamin B1 expression in adult neurogenesis (Figure 11B), indicating that changes in lamin B1 protein sequences may contribute to the changes in lamin B1 expression patterns.

Since PTMs regulate the stability of lamin B1 protein, we next assessed the conservation of PTM sites in lamin B1 among experimental animals. It is generally assumed that the conservation of an amino acid site can serve as a good approximation for the conservation of PTMs at that site.^62^ Therefore, we focused on PTMs reported in mouse lamin B1 protein (Uniprot, PhosphoSite Plus, and iPTM) and considered the PTM sites conserved if the respective amino acid residues were present at the same positions in other species. Our PTM analysis revealed that out of 57 reported PTMs in mouse lamin B1, 35 are fully conserved across the six species. Among them are the well described phosphorylation mitotic site motifs S24 and S394, which are required for mitotic nuclear envelope breakdown (Figure 12A).^63^, ^64^ On the other hand, 22 PTM sites were not conserved across six species, and six PTMs were specifically not conserved between mammalian and anamniote groups (Figure 12A, highlighted in red), including K80 and K103 ubiquitination, S159, T284, and S376 phosphorylation, as well as K168 acetylation. Although the functional significance of these non‐conserved PTM sites on lamin B1 function remains to be determined, differences in PTMs imply distinct modifications of lamin B1 proteins between mammals and anamniotes.

Taken together, our analyses indicate that lamin B1 protein sequence similarity correlates with differences in lamin B1 expression patterns. Further experiments addressing the functional implications of these differences will contribute to our understanding of the role of lamin B1 in the maintenance of adult neurogenesis across evolutionary scales.

## 3 DISCUSSION

### 3.1 Different patterns of lamin B1 expression between mammals and anamniotes

Previous studies have shown that nuclear structural proteins drive cell‐type‐specific nuclear architecture, regulating epigenetic programs and maintaining cell‐type‐specific functions,^32^, ^65^, ^66^ including the maintenance of somatic stem cells and their capacities. In the context of adult neurogenesis, lamin B1 has been highlighted for its role in maintaining adult neurogenesis in mice.^30^, ^31^ Since neurogenic capacity decreases significantly from anamniotes to higher amniotes,^3^, ^4^ we examined whether the expression pattern of lamin B1 is also changed or conserved across species. Our results from three different mammals with distinct lifespans indicate that the expression pattern of lamin B1 during adult neurogenesis is well conserved in two neurogenic regions within mammals. In mammals, neural stem cells and neuroblasts exhibited higher lamin B1 levels and differentiated neurons possessed lower lamin B1 levels (Figures 11 and 12). On the other hand, three anamniotes showed distinct patterns of lamin B1 expression from mammals. In larger, longer‐lived species, proliferation rates and maturation times differ from those in mice. Comparative analyses have revealed that longer‐lived species take longer to reach maturity, produce fewer new cells in the adult brain, but continue cell proliferation later in life.^67^, ^68^, ^69^ Therefore, the lifespan of a model species is a critical factor in making comparative inferences. Intriguingly, despite the difference in lifespan and rate of maturity among the studied species, the expression patterns of lamin B1 were conserved within mammals, highlighting the potential universal importance of lamin B1 in maintaining ANSPC populations and regulating neuronal differentiation.

For the maintenance of neurogenic capacity, the studies from the mouse DG revealed that high lamin B1 levels in ANSPCs inhibit differentiation from ANSPCs, while a reduction of lamin B1 induces neural differentiation,^30^, ^31^ indicating the functional role of lamin B1 in maintaining ANSPCs. Mechanistically, to maintain ANSPCs, lamin B1 interacts with chromatin and represses developmental genes in mouse NSPCs.^30^ Then, why are lamin B1 levels in ANSPCs low in salamanders and fish, even though they show a higher neurogenic rate and regenerative capacity? One possibility is that lower lamin B1 levels in ANSPCs may help to maintain ANSPCs in a “permissive state” for rapid proliferation/differentiation when needed. In this case, ANSPCs in anamniotes may require additional epigenetic mechanisms to prevent the depletion of ANSPCs. Future research should explore epigenomic regulation in the context of lamin B1 involvement in salamanders and zebrafish.

It is interesting to note that the levels of lamin B1 are higher in the SVZ than the DG in mammals. Adult neurogenesis in the SVZ involves long‐distance migration of neuroblasts, making them more vulnerable to nuclear damage during migration. Since lamin B1 protects nuclei from damage during migration,^45^, ^70^ higher levels of lamin B1 might be beneficial for promoting chromatin integrity or neuronal survival.

### 3.2 Inversed patterns of lamin B1 expression in the neurogenic trajectory during evolution

A previous report suggested that lamin B1 levels vary across vertebrate evolution. It has been observed that lamin A expression is low in cartilaginous and ancient bony fish and increases towards mammals, while lamin B1 demonstrates an inverse trend across various tissues such as brain, heart, kidney, liver, testis, and ovary.^71^ Consistent with this idea, the relative levels of lamin B1 in mature neurons are low in mammals, yet high in anamniotes. However, our observation in the neurogenic niche indicates the opposite patterns in terms of lamin B1 expression. This suggests that the mechanisms controlling the expression of the *lmnb1* gene in the brain, especially in the neurogenic niche, have significantly changed from anamniotes to mammals. Furthermore, it is possible that the function or stability of lamin B1 protein may have been altered during evolution. Indeed, about 30% of amino acid sequences of lamin B1 proteins diverge between mammals and anamniotes (Figure 11A). Although the major structural features of lamin B1, such as the head, coiled‐coil domains with linkers, tail domain with an IG‐like fold, NLS, and CaaX motif, are conserved across the studied species, changes in specific amino acid sequences including PTM sites may significantly impact its stability and function. Lamin B1 interacts with various other proteins including other lamins, nucleoporins, histones, emerin, BANF1,^72^, ^73^ HDAC1,^74^ and SUN1.^75^ The interaction with those proteins is crucial for lamin B1 function, but its significance is usually investigated in mammalian cells. In the future, to understand the difference of neurogenic capacity between mammals and anamniotes, it would be important to address the difference of lamin B1 co‐factors between mammals and anamniotes. Intriguingly, in addition to long‐lived stable proteins, it was also recently found that some of nuclear RNAs are also extremely stable in mammalian ANSPCs,^76^ and they are likely to interact with the nuclear periphery. Therefore, it would be of great interest to understand how long‐lived proteins such as lamin B1 and long‐lived RNAs interact physically and functionally during evolution. Furthermore, there is a general shift during evolution from stem cell dependent regenerative plasticity to circuit refining plasticity.^77^ It would be interesting to address whether these long‐lived cellular complements contribute to this evolutionary shift.

## 4 EXPERIMENTAL PROCEDURES

### 4.1 Animals and brain tissue processing

#### 4.1.1 Mouse

All procedures relating to the mouse care and treatment were approved by the Government of Saxony and performed in accordance with their guidelines. To measure the levels of lamin B1 in the brain, we used 3 male C57BL/6 mice with an age of 3 months. Mice were euthanized by a lethal dose of sodium‐pentobarbital (WDT). After confirming the death of the animals, the animals were transcardially perfused with ice‐cold 1× phosphate‐buffered saline (PBS), followed by 50 mL of freshly prepared 4% paraformaldehyde (PFA, Sigma Aldrich) in PBS. Brains were removed from the skull and post‐fixed for 24 h in 10 mL 4% PFA at 4°C. Afterwards, the brains were washed twice with 1× PBS and transferred into 15% sucrose in PBS. After an incubation of one night, the brains were incubated in 30% sucrose in PBS for two overnights at 4°C for cryoprotection. After the 40 μm thick coronal sections were made using a sliding microtome (Leica SM 2010 R), the sections were stored in cryoprotective solution (25% ethylenglycol (Roth), 25% glycerol (VWR) in 0.1 M phosphate buffer, pH 7.4) at −20°C.

#### 4.1.2 Naked mole‐rat

Adult naked mole‐rats (NMR) (*Heterocephalus glaber*) were obtained at Kumamoto University and Dresden Zoo in accordance with their guidelines. The sacrificing and tissue processing of the naked mole‐rat were performed as described above. The paraffin‐embedded NMR brain tissues from three animals were prepared as described previously.^78^

#### 4.1.3 Ferret

A pigmented ferret (*Mustela putorius furo*) was obtained from Euroferret and kept at the Animal Facilities of the Universidad Miguel Hernández. All animals were treated according to Spanish and EU regulations, and experimental protocols were approved by the Universidad Miguel Hernández Institutional Animal Care and Use Committee (IACUC).

#### 4.1.4 Axolotl and Newt

Adult *Pleurodeles waltl* and sexually mature *Ambystoma mexicanum* were raised in a colony at CRTD, TU Dresden. All procedures relating to axolotl and newt's care and treatment were approved by the Government of Saxony and performed in accordance with their guidelines. All animals were kept in the animal facility under standard conditions of 12 h light/12 h darkness at 18–24°C. Animals were terminated by anesthesia overdose, and brains were dissected out and fixed in 4% PFA in PBS at 4°C overnight. Then tissues were dehydrated, embedded in paraffin (STP 420D) and subsequently sectioned to 10 um‐thick slices on a microtome and stored at 4°C for later use.

#### 4.1.5 Zebrafish

Wild‐type zebrafish from the AB genetic background used in this study were raised and maintained as described previously.^79^ Adult fish, 6 months of age, were euthanized by rapid chilling through submersion in an ice water bath for 5 min. The fish brains were subsequently dissected in PBS and fixed overnight in 4% formaldehyde within a phosphate buffer (0.1 M, pH 7.5). Following fixation, the brains were transferred to a cryo‐protection solution comprising 30% sucrose in phosphate buffer, with the solution being refreshed daily for 2 days. The brains were then embedded in OCT (Tissue Tek) and frozen in cryomolds using dry ice. After overnight storage at −80°C, the brains were sectioned into 14 μm slices using the CryoStar NX70 Cryostat (Thermo Scientific), collected onto Superfrost Plus Gold (Thermo Scientific) microscope slides, dried at 55°C for 2 h, and stored at −20°C.

### 4.2 Immunohistochemical staining procedures

For immunohistochemical staining of all experimental brain tissues, the used antibodies with their respective concentrations are shown in Table 1.

**TABLE 1:** List of the used antibodies.

Antibody	Host/Isotope	Dilution	Distributor, Cat. number	Target species
Primary antibodies				
Lamin B1	Rabbit, IgG polyclonal	1:2000	Abcam, ab16048	Mouse, naked mole‐rat, ferret, axolotl, newt, zebrafish
Sox2	Rat, monoclonal IgG2a	1:2000	ThermoFisher, 14‐9811‐82	Mouse, naked mole‐rat, ferret
GFAP	Goat, polyclonal	1:1000	Abcam, ab53554	Axolotl, newt
GFAP	Mouse, monoclonal IgG1	1:500	Zfin, ZRF‐1	Zebrafish
DCX	Goat, polyclonal IgG	1:250	Santa Cruz Biotec, sc‐8067	Mouse, naked mole‐rat, ferret
Prox1	Mouse, monoclonal IgG	1:250	Millipore, MAB5654	Mouse
NeuN	Mouse, monoclonal IgG1	1:250	Millipore, MAB377	Mouse, naked mole‐rat, ferret
Huc/D	Mouse, IgG2b	1:300	Invitrogen, A‐21271	Zebrafish
Ctip2	Rat	1:200	Abcam, ab18465	Axolotl, newt
Secondary antibodies				
Anti‐Rabbit‐Cy3	Donkey IgG	1:500	Jackson IR, 711–165‐152	Mouse, naked mole‐rat, ferret, axolotl, newt, zebrafish
Anti‐Rat‐Cy5	Donkey IgG	1:500	Jackson IR, 712–175‐153	Mouse, naked mole‐rat, ferret, axolotl, newt
Anti‐Mouse‐A488	Donkey IgG	1:500	Jackson IR, 715–545‐150	Mouse, naked mole‐rat, ferret, zebrafish
Anti‐Goat‐A488	Donkey IgG	1:500	Dianova, 705–545‐147	Mouse, naked mole‐rat, ferret
Anti‐Goat‐A647	Donkey IgG	1:500	Dianova, 705–605‐147	Axolotl, newt
DAPI		1:2000	ThermoFisher, 1,387,190	Mouse, naked mole‐rat, ferret, axolotl, newt, zebrafish

The protocols for IHC staining and related procedures were optimized specifically for each species and are presented in separated steps below.

#### 4.2.1 IHC procedures for free‐floating sections (mouse, ferret)

For the IHC staining, free‐floating sections were washed in 2 mL washing buffer (1× PBS with 0.2% Triton X‐100) three times for 15 min. Sections were blocked with a washing buffer supplemented with 3% horse serum (VWR international) (blocking buffer) for 1 h at room temperature. Then, the sections were incubated with primary antibodies for 48 h at 4°C on a shaker. After the incubation, the sections were washed and incubated with the secondary antibodies and DAPI for 2 h at room temperature. After the incubation with the secondary antibodies, the sections were finally washed three times and mounted on a glass slide. 100 μL of Mowiol with 2.5% DABCO (Sigma) was used as mounting media.

#### 4.2.2 IHC procedures with the paraffin‐embedded sections (NMRs, salamanders)

For the paraffin embedded NMR and salamander tissues, the additional deparaffinization and hydration steps were applied. For removing paraffin, slides with samples were incubated in xylene for 10 min twice, followed by an ethanol series to hydrate the tissues. For that, tissues were incubated twice in 100% ethanol for 10 min each, then in 96%, 80%, 70%, and 50% for 2 min each, and finally incubated in distilled water for 10 min. Then heat‐induced antigen retrieval was performed. Citrate buffer (10 mM, pH 6.0) was used. The tissues were placed in a chamber with pre‐heated citrate buffer and incubated for 20 min at 98°C in a steamer. After cooling down to room temperature, tissues were rinsed in the washing buffer three times and were incubated in a blocking solution for 1 h, followed by IHC staining described above for mouse tissues.

#### 4.2.3 Zebrafish IHC staining procedures

For OCT embedded fixed tissues of the zebrafish brain, the slides with samples were first washed three times in PBS and subsequently incubated in a hot citrate buffer as described for the antigen retrieval above. The IHC stainings for OCT zebrafish sections were performed as described above for other species.

### 4.3 Image acquisition

Fluorescence staining was imaged in 3D using LSM 980 (Zeiss) or Axio observer Z1 (Zeiss). For the acquisition of images for lamin B1 intensity measurements, the LSM 980 Airy Scan (Zeiss) with the ZEN 3.3 software was used. Within the same species, the same imaging condition was applied.

### 4.4 Image analysis

The image analysis was performed with Fiji as described previously.^30^ Briefly, the background intensity of images was measured outside the DG, SVZ, or pallium, drawn with the rectangle selection. The averaged intensity of the background was subtracted. The center of nuclei was determined based on the DAPI signal. The intensity of lamin B1 was determined based on the signals at the nuclear periphery as described previously.^30^

### 4.5 Protein sequence comparison

#### 4.5.1 Sequence retrieval

Lamin B1 protein sequences were either retrieved from NCBI (http://www.ncbi.nlm.nih.gov), UniProt (uniprot.org), Ensembl (http://www.ensembl.org) or Axobase (https://www.axobase.org). To retrieve the sequence of newt lamin B1, we used BLASTp with a protein database for iNewt (https://www.nibb.ac.jp/imori/main/?page_id=8), since it was not present in open databases. The lamin B1 sequence of axolotl was used as a query sequence.

#### 4.5.2 Protein alignment, protein analysis, and phylogenetic reconstruction

Protein sequences were aligned using UGENE software with the Clustal Omega algorithm. To annotate the lamin B1 regions and motifs, NCBI and UniProt databases were used. Sequence identity and similarity values were calculated using the Ident and Sim function of the sequence manipulation suite (http://www.bioinformatics.org/SMS/index.html) with the following groups of similar amino acids for the similarity calculations: ILV, FWY, KRH, DE, GAS, P, C, or TNQM. These abbreviations represent groups of amino acids that share similar physicochemical properties or are often conserved together due to their structural or functional roles in proteins. They are commonly used as default settings for multiple sequence alignment (MSA).^80^ Evolutionary analysis was conducted in MEGA11. The phylogenetic tree of studied species was created based on lamin B1 protein sequences. The evolutionary history was inferred by using the Maximum Likelihood method and JTT matrix‐based model.^81^ The tree with the highest log likelihood (−2939.00) is shown. Branch lengths indicate genetic change; that is, the longer the branch, the more genetic change (or divergence) has occurred.

### 4.6 Statistics

Statistics was performed using GraphPad Prism 9 and Excel (Microsoft). Normality was tested by the Shapiro–Wilk test. *p*‐Values were determined by unpaired Student's *t*‐test, a one‐sample *t*‐test, a Mann–Whitney *U*‐test, Kruskal–Wallis test, or one‐way ANOVA as indicated. All values are presented as mean ± SEM.

## CONFLICT OF INTEREST STATEMENT

The authors declare no conflicts of interest.
